# Precise Layer Control of MoTe_2_ by Ozone Treatment

**DOI:** 10.3390/nano9050756

**Published:** 2019-05-17

**Authors:** Qiyuan Wang, Jing Chen, Youwei Zhang, Laigui Hu, Ran Liu, Chunxiao Cong, Zhi-Jun Qiu

**Affiliations:** State Key Laboratory of ASIC & System, School of Information Science and Technology, Fudan University, Shanghai 200433, China; 14110720018@fudan.edu.cn (Q.W.); 17110720037@fudan.edu.cn (J.C.); 12110720052@fudan.edu.cn (Y.Z.); laiguihu@fudan.edu.cn (L.H.); rliu@fudan.edu.cn (R.L.)

**Keywords:** MoTe_2_, ozone, self-limiting, layer thinning

## Abstract

Transition metal dichalcogenides (TMDCs) demonstrate great potential in numerous applications. However, these applications require a precise control of layer thickness at the atomic scale. In this work, we present an in-situ study of the self-limiting oxidation process in MoTe_2_ by ozone (O_3_) treatment. A precise layer-by-layer control of MoTe_2_ flakes can be achieved via multiple cycles of oxidation and wet etching. The thinned MoTe_2_ flakes exhibit comparable optical properties and film quality to the pristine exfoliated ones. Besides, an additional p-type doping is observed after O_3_ oxidation. Such a p-doping effect converts the device properties of MoTe_2_ from electron-dominated to hole-dominated ambipolar characteristics.

## 1. Introduction

The past decade has witnessed a rapid development in two-dimensional (2D) materials research with a focus on the family of transition metal dichalcogenides (TMDCs) [1,2,3]. Depending on polytype and the number of transition metal d-electrons, TMDC materials exhibit a wide range of electronic properties, from semiconducting, metallic, to superconducting [1]. These unique electrical and optical properties are highly thickness dependent. Two-dimensional materials like MoS_2_, MoSe_2_, and their tungsten analogs, with their indirect bandgap transformed to direct in the monolayer limit [4,5,6], could be used as promising candidates for applications in electronics and optoelectronics [7,8,9].

The direct bandgap depends on the chalcogen species chosen in TMDCs. Compared with other TMDC materials, monolayer MoTe_2_ has a direct bandgap of 1.1 eV, which is the smallest among all semiconducting TMDCs [10,11]. MoTe_2_ is thought to be an ideal candidate to bridge large-bandgap TMDCs and gapless graphene. Because its bandgap is comparable to that of silicon, MoTe_2_ can potentially expand the range of TMDC optoelectronic applications beyond the visible spectrum. In contrast to most TMDCs that exhibit indirect bandgaps in multilayer/bulk forms, MoTe_2_ still preserves its direct bandgap feature in bilayers, and perhaps even in trilayers with less change in bandgap value [12]. Furthermore, the narrow bandgap of MoTe_2_ facilitates the construction of n- and p-type transistors due to low Schottky barrier heights (SBHs) for electrons and holes [13]. In this regard, field effect transistors (FETs) built from MoTe_2_ have been demonstrated to display n-type, p-type, and ambipolar behaviors [14,15,16,17,18]. 

Since the electronic properties of 2D materials are highly dependent on the layer number, a simple and efficient method to precisely control layer thickness is a prerequisite for various applications. So far, several strategies have been established to prepare 2D TMDCs with a certain layer thickness, which can be generally categorized into top-down and bottom-up technologies. The top-down methods normally involve mechanical [19] and solution-based exfoliation [20] from bulk materials. Exfoliation-based techniques are very common, but typically generate flakes with random sizes and thicknesses. On the other hand, the bottom-up method usually yields 2D materials via chemical reactions of atoms/molecules on a substrate with a large power consumption [21]. 

In comparison, the post-treatment of multilayer materials is an alternative route to accurately control the layer number. Various layer-thinning technologies have been conducted, such as laser thinning [22], thermal annealing [23], and plasma etching [24]. TMDCs are known to potentially oxidize in ambient environments. This oxidation process is self-limiting to some degree, since the surface oxide film hinders oxygen diffusion into the underlying layers [25]. However, higher operating/processing temperatures enhance oxygen diffusion and accelerate the oxidation process. This leads to more layers being oxidized simultaneously and to lack a precise control over the number of layers. Hence, a fast reaction between the oxidizing agents and the surface layer, accompanied by a slow diffusion rate to the underlying layers, is expected to be a promising route to achieve controlled layer-by-layer thinning at the atomic scale. Compared with other oxidizing agents, ozone (O_3_) is a strong oxidant, which is unstable and easily decomposes into molecular O_2_ and monatomic O when reaching a solid surface; the latter is regarded as a main active species that vigorously reacts with TDMCs or other 2D materials [26,27,28]. Herein, we performed an in-situ study of O_3_ exposure of MoTe_2_ to monitor the evolution of layer number and characterize the quality of the thinned layers. A precise layer-by-layer control of MoTe_2_ layers was achieved via the cyclical processing of oxidation and subsequent removal of the oxidized layer. The thinned MoTe_2_ flakes exhibited comparable optical properties to the pristine exfoliated ones, and showed a p-type doping behavior.

## 2. Materials and Methods 

MoTe_2_ flakes were mechanically exfoliated from bulk 2H-MoTe_2_ crystals (2D Semiconductors, Inc., Scottsdale, AZ, USA) and then deposited on a heavily p-doped Si substrate with a 300-nm-thick oxide layer. The thickness of the MoTe_2_ flakes was first identified using an optical microscope (VHX-600, Keyence, Inc., Itasca, IL, USA) through optical contrast and then further confirmed by atomic force microscopy (AFM, Dimension 3100, Veeco, Inc., New York, NY, USA) and Raman spectroscopy (Renishaw inVia, Renishaw, Inc., Gloucestershire, UK). Raman spectra were collected in a backscattering geometry with a 633 nm laser, 100× objective, and 1800 lines/mm grating. The laser power was kept below 0.1 mW to avoid sample damage. 

Afterward, MoTe_2_ flakes were placed in a custom-designed container for in-situ Raman investigation. O_3_ was remotely generated by electric discharge with an O_3_ generator (M-600, Tonglin, Inc., Beijing, China) at an O_2_ pressure of 0.3 bar and then introduced to the chamber. The oxidized samples were immersed in KOH solution to remove the surface oxide and washed with deionized water. The chemical states of the MoTe_2_ specimen before/after O_3_ oxidization and after KOH treatment were characterized by X-ray photoelectron spectroscopy (XPS, Thermo Scientific Escalab 250Xi, Thermo Fisher Scientific, Inc., Waltham, MA, USA) using a monochromatic Al Kα X-ray source with a 100 μm spot size. 

For electrical characterization, a back-gated MoTe_2_ FET device was fabricated on a SiO_2_/Si substrate. Multilayer MoTe_2_ was first transferred onto the substrate. The electrode patterns were defined by standard electron-beam lithography (JBX-6300FS, JEOL, Inc., Tokyo, Japan). A bilayer stack of Cr (5 nm)/Au (50 nm) was deposited by thermal evaporation on the flake to form source/drain electrodes. A lift-off process was then performed in acetone and isopropanol to complete the device fabrication. Multiple cycles of O_3_ oxidation and oxide layer removal were repeated on the MoTe_2_ channel to achieve layer-by-layer thinning. After each thinning cycle, the same device was characterized with a semiconductor parameter analyzer (B1500A, Agilent Inc., Santa Clara, CA, USA).

## 3. Results and Discussion

**Figure 1:**
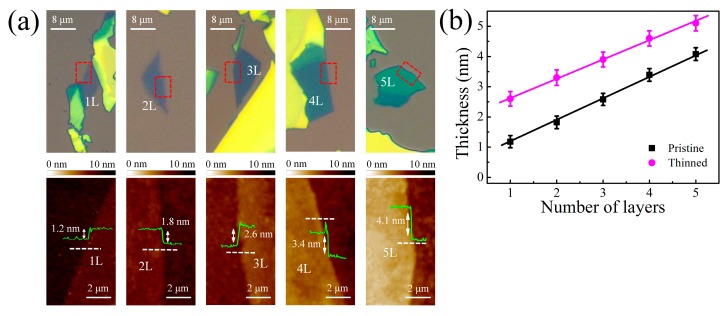
(**a**) Optical images and the corresponding AFM images of monolayer (1L) to pentalayer (5L) pristine MoTe_2_ flakes on SiO_2_/Si substrate. The regions enclosed by the red dashed lines indicate the AFM scanned areas. The green curves are AFM height profiles taken along the white dashed lines on the AFM images. (**b**) Plots of thickness of pristine and thinned MoTe_2_ versus layer number. Solid lines are linear fits.

**Figure 2:**
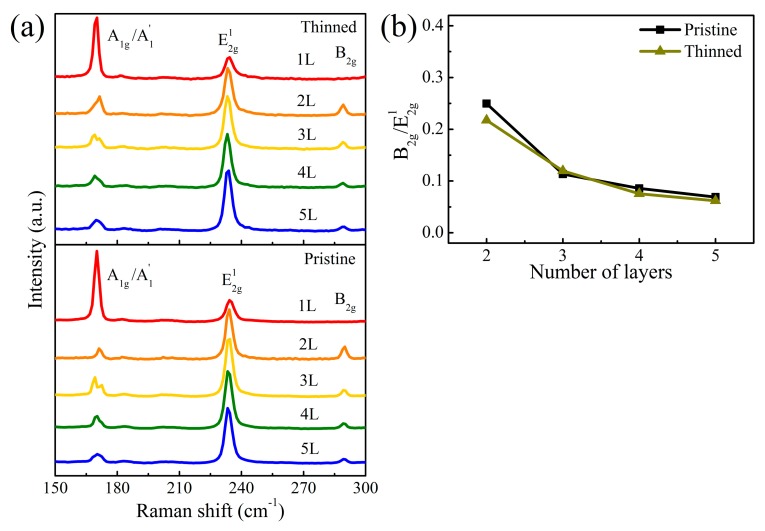
(**a**) Raman spectra for pristine and thinned MoTe_2_. All spectra are offset vertically for clarity. (**b**) The relationship between peak intensity ratio of B_2g_/E_2g_^1^ and the layer number for pristine and thinned MoTe_2_.

Figure 1a shows the optical microscope images and the corresponding AFM images of pristine MoTe_2_ with different layer numbers varying from monolayer to pentalayer. The number of layers and the corresponding thickness were first estimated from optical contrast and then further determined by AFM and Raman scattering. Obviously, the optical contrast of MoTe_2_ increases with the layer number due to the interference effect [29]. AFM height profiles of different layers are superimposed in the AFM images. A linear dependence of the layer thickness is observed with the layer number in Figure 1b. The interlayer distance was approximately 0.7 nm, which is in good agreement with the bulk interlayer spacing of MoTe_2_ [30]. Figure 2a shows the Raman spectra of MoTe_2_ flakes with several characteristic peaks between 150 and 300 cm^−1^. The peaks located at ~170, 230, and 290 cm^−1^ correspond to the out-of-plane mode A_1g_/A_1_^’^ (A_1_^’^ for odd layers and A_1g_ for even layers), the in-plane mode E_2g_^1^, and the bulk-inactive mode B_2g_, respectively [31,32]. The B_2g_ peak also vanished in monolayer (1L) but became active in few-layer MoTe_2_ due to translation symmetry breaking [33]. This can be a landmark to distinguish monolayers from few layers. Furthermore, the intensity of the B_2g_ peak was sensitive to the thickness. As shown in Figure 2b, the peak intensity ratio of B_2g_ to E_2g_^1^ consistently decreased with the layer number. This ratio can therefore be used to identify the number of MoTe_2_ layers, like the frequency difference between the A_1g_ and E_2g_^1^ modes utilized in MoS_2_. Different from MoS_2_, the interlayer interactions also caused a Davydov splitting of A_1g_/A_1_^’^ modes in MoTe_2_ at thicknesses larger than bilayer [34].

**Figure 3:**
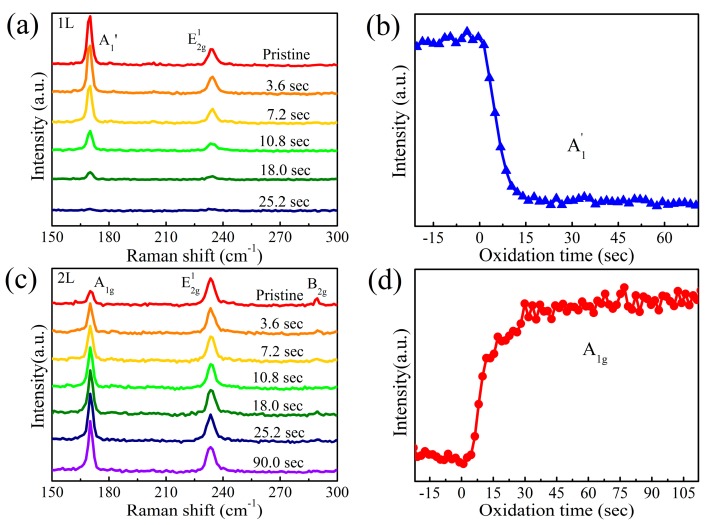
Raman spectral evolution of (**a**) 1L and (**c**) 2L MoTe_2_ during O_3_ exposure. Raman intensities of (**b**) A_1_^’^ and (**d**) A_1g_ modes as a function of oxidation time.

**Figure 4:**
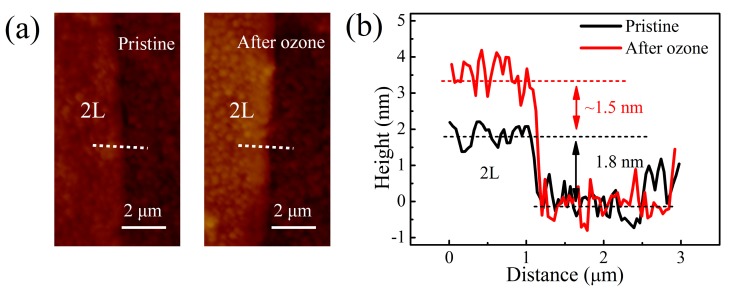
(**a**) AFM images of 2L MoTe_2_ flake squared in Figure 1a at pristine state (left) and after O_3_ treatment (right). (**b**) AFM height profiles of pristine (black line) and oxidized (red line) 2L MoTe_2_ along the white dashed lines in (a).

After carefully characterizing the pristine exfoliated MoTe_2_, an in-situ Raman study of O_3_ exposure was performed to monitor the time evolution of the Raman spectra of MoTe_2_ during oxidation. First, a monolayer MoTe_2_ surface was exposed to the O_3_ for 70 s. As shown in Figure 3a,b, the peak intensities of the A_1_^’^ and E_2g_^1^ modes decreased with exposure time and completely disappeared in 25 s. The absence of Raman signals after the O_3_ treatment indicates that MoTe_2_ was fully oxidized. In contrast, under the same oxidation conditions, the A_1g_ peak of bilayer MoTe_2_ became more prominent relative to the E_2g_^1^ mode with oxidation time (Figure 3c). Simultaneously, the characteristic B_2g_ peak gradually disappeared with only A_1g_ and E_2g_^1^ modes remaining. This suggests that bilayer MoTe_2_ was transformed into monolayer. Furthermore, the A_1g_ peak intensity reached a maximum and remained nearly invariant after 25 s oxidation time (Figure 3d). However, the O_3_ oxidation of MoTe_2_ could proceed if UV illumination were introduced [28]. This hints that pure O_3_ exposure leads to a dense coverage of surface oxide layer on atomically thin MoTe_2_. The AFM images and the height profiles in Figure 4 show that the bilayer thickness increased by 1.5 nm after oxidation. This surface oxide layer suppressed the further diffusion of the oxygen atoms into the flake and their oxidation of the underlying layers, even with longer exposure times. This oxidation process was therefore self-limiting. Notably, the time to fully oxidize the monolayer and the top layer of MoTe_2_ flakes was almost the same, irrespective of layer numbers.

**Figure 5:**
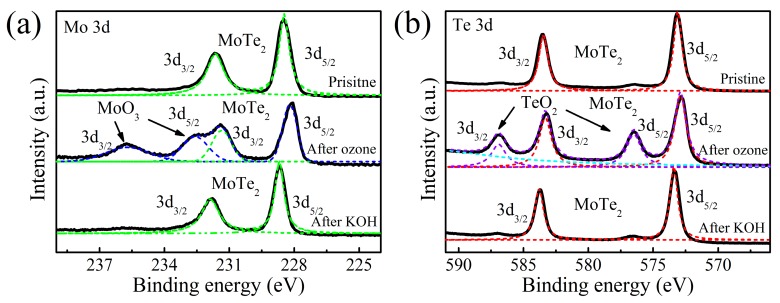
XPS spectra of (**a**) Mo 3d and (**b**) Te 3d core levels of MoTe_2_ before/after O_3_ oxidation and after KOH treatment. The black curves are experimental data. The dashed curves are the Lorentzian fits for the peaks of MoTe_2_, MoO_3_, and TeO_2_, respectively. The spectra are offset vertically for clarity.

In order to realize a controllable layer-by-layer thinning of multilayer flakes, the surface oxide layer in MoTe_2_ needs to be removed and the underlying layer freshly exposed to provide a new surface to be reacted with O_3_. Generally, base (OH−) solutions are good solvents for metal oxides. Herein, an oxidized thick MoTe_2_ sample was immersed in KOH solution followed by a deionized water rinse. Afterward, XPS analysis was conducted on the MoTe_2_ to investigate the evolution of the surface chemical compositions before and after KOH solution immersion. As shown in Figure 5a, two dominant peaks in pristine MoTe_2_ were assigned to the Mo^4+^ 3d_3/2_ (231.7 eV) and 3d_5/2_ (228.5 eV) [35]. Te^2−^ 3d_3/2_ and 3d_5/2_ were located at 583.5 and 573.2 eV, respectively (Figure 5b). However, after O_3_ treatment, the binding energies of Mo^4+^ and Te^2−^ peaks were both red-shifted by approximately 0.4 eV. Meanwhile, two new peaks appeared at 235.7 and 232.6 eV, belonging to the Mo^6+^ 3d_3/2_ and 3d_5/2_ doublet of MoO_3_ [35,36]. This similar phenomenon was also found in Te 3d core level spectra. Two emerging peaks at 586.9 and 576.5 eV were associated with TeO_2_ formation (Figure 5b). The shift of binding energy is related with the Fermi level energy realignment [37]. The coverage of high work function Mo and Te oxides aligns the Fermi level energy close to the valence band edge of MoTe_2_ and thus induces redshifts of Mo^4+^ and Te^2−^ peaks and p-type doping in the underlying MoTe_2_ [38]. After KOH solution immersion, the XPS peaks related with oxides vanished and the Mo^4+^ and Te^2−^ peaks shifted back close to the original pristine positions. This suggests that most of MoO_3_ and TeO_2_ were removed and the p-doping effect was reduced. 

**Figure 6:**
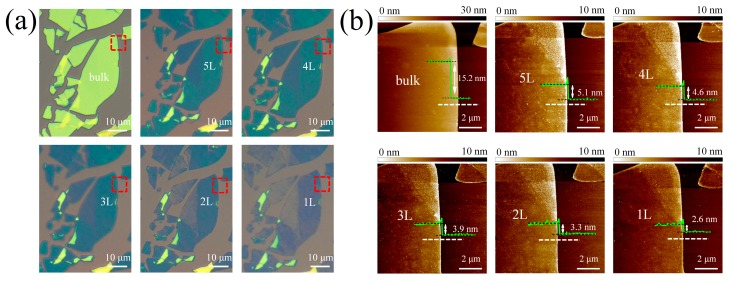
(**a**) Optical images of large-area MoTe_2_ flakes before and after thinned from bulk to 5L, 4L, 3L, 2L and 1L MoTe_2_. (**b**) The corresponding AFM images of the selected square regions in (a), with superimposed height profiles along the dashed lines.

We repeated multiple oxidation/oxidized layer removal cycles to perform a layer-by-layer thinning process in large-area thick MoTe_2_ flakes (Figure 6a). Obviously, the optical contrast of MoTe_2_ flakes changed significantly after 24 cycles. One cycle corresponded to one layer removal. Figure 6b shows the AFM images and height profiles of the selected areas in Figure 6a. The relationship between the layer thickness and the layer number is plotted in Figure 1b. Obviously, a linear dependence was also found and the slope was the same as that of pristine MoTe_2_. This further confirms a precise control of the layer number at the atomic scale by using a cyclical thinning process. The surface roughness of thinned flakes was almost comparable to that of the pristine ones, with only a slight increase from 0.2 to 0.25 nm. However, the thinned layer thickness showed an overall upshift of 1.4 nm. Such a shift may originate from the water molecules trapped between the flakes and the substrate in the wet process [39]. Figure 2 also illustrates comparable Raman spectra between the thinned and pristine MoTe_2_, and almost the same peak intensity ratio B_2g_/E_2g_^1^.

**Figure 7:**
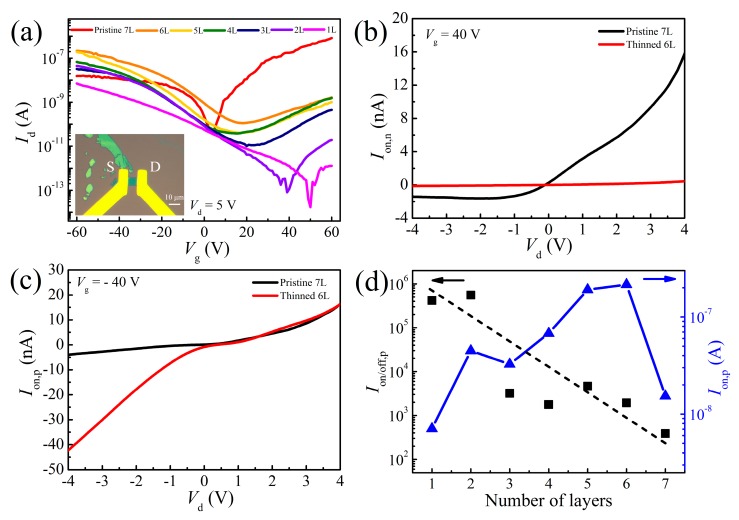
(**a**) Transfer characteristics of a 7L MoTe_2_ field effect transistor (FET) before and after layer-by-layer thinning. The inset is an optical image of the as-fabricated device. (**b**,**c**) Comparison of the output characteristics in the n- and p-type regimes before and after layer thinning. (**d**) *I*_on/off,p_ (left) and *I*_on,p_ extracted at *V*_g_ = −60 V in (a) (right) plotted as a function of the number of MoTe_2_ layers. The dashed line is a guide to the eye.

To study the effect of layer thinning on the electrical characteristics of the MoTe_2_ layers, a back-gated FET device was fabricated on a 300-nm SiO_2_/Si substrate. The inset of Figure 7a presents an optical image of a device with seven pristine layers in the channel. The electrical behaviors were investigated while thinning the MoTe_2_ from seven layers to monolayer by using the cyclical thinning method. Figure 7a shows the evolution of the transfer characteristics with different layer numbers. The source–drain voltage was fixed at 5 V. Before thinning, a typical electron-dominated ambipolar behavior was observed in pristine exfoliated layers, where the on-state current in the n-branch (*I*_on,n_) was one order of magnitude larger than that in the p-branch (*I*_on,p_). This asymmetric conduction behavior was attributed to the unequal SBHs for electrons and holes. This was manifested by the non-linear output characteristics measured at *V*_g_ = ±40 V in Figure 7b,c. The vacancies were found to be easily formed in MoTe_2_ due to the weak bonding energy between Mo and Te atoms [40]. The presence of chalcogen vacancies in TMDCs usually causes the Fermi level to pin near the conduction band [41,42]. Consequently, the electron SBH is lower than that of the holes, thus facilitating the electron injection. Due to a smaller bandgap in the thick pristine MoTe_2_, the current on/off ratio (*I*_on/off_) over the applied gate voltage range was quite small, 2.1 × 10^4^ for n-branch and 3.9 × 10^2^ for p-branch. After cyclical process of O_3_ oxidation and subsequent oxide removal, *I*_on,n_ was found to be significantly suppressed, as exhibited in Figure 7b and it reduced with decreasing layer number (Figure 7a). Meanwhile *I*_on,p_ was drastically increased by one order of magnitude after the first cycle (Figure 7d), that is, in the thinned 6L MoTe_2_, and then gradually declined with decreasing thickness. Simultaneously, *I*_on,p_ became more linear in the output characteristics, which means a reduced hole SBH formed after the thinning process. A remarkable increase of *I*_on/off_ in the p-type regime (*I*_on/off,p_) was observed with decreasing layer number. This was mainly attributed to the suppression of off-state current from electron conduction. These distinct transport properties between the pristine and O_3_ treated MoTe_2_ were possibly caused by the residues of overlying oxide after KOH immersion. The above XPS analysis reveals that MoO_3_ and TeO_2_ contributed to p-doping on the underlying MoTe_2_ layers. The hole carriers residing between the MoTe_2_ and contact can unpin the Fermi level and lower it toward the valence band edge [38]. Hence, the reduced hole SBH, along with the raised electron SBH made a transition of the dominant role from electron to hole in the ambipolar MoTe_2_ FET. Meanwhile, as shown in Figure 7a, the oxide doping also shifted the voltage of the charge neutral point towards the positive direction with thinning of the MoTe_2_ layers. After the first thinning cycle, *I*_on,p_ exhibited a decline with decreasing layer number in Figure 7d, which is consistent with previous observations on MoS_2_ and black phosphorus [43,44]. The conduction paths along the upper layers were eliminated when these layers were removed during the thinning process. An additional access resistance was hence introduced between the electrode and the underlying MoTe_2_ due to the large interlayer resistance. This access resistance increased as the layers were thinned. Besides, another effect may exist—thinner flakes are more susceptible to Coulomb scattering from the doping species, which leads to a reduction of carrier mobility [45]. 

## 4. Conclusions

In summary, we demonstrated a controllable layer thinning of MoTe_2_ flakes with O_3_ treatment. Our in-situ Raman investigation revealed that the formed oxide layer on the surface of MoTe_2_ led to a self-limiting process. This process could be repeated by removal of the oxidized surface to achieve precise layer-by-layer thinning. The thinned MoTe_2_ flakes showed a comparable optical quality and surface roughness to the pristine exfoliated ones. This thinning process was also accompanied by a p-type doping in the MoTe_2_ flakes due to oxide layer coverage. The device transfer characteristics of the MoTe_2_ FET exhibited a conversion from electron-dominated to hole-dominated ambipolar behavior as the thinning cycle increased. The *I*_on/off_ was thus significantly increased at the p-branch. It is believed that our cyclical thinning technique can be applicable to other TMDCs, and provides excellent control in preparing TMDC sheets with well-defined thickness.
